# Antifungal Drug Repurposing

**DOI:** 10.3390/antibiotics9110812

**Published:** 2020-11-15

**Authors:** Jong H. Kim, Luisa W. Cheng, Kathleen L. Chan, Christina C. Tam, Noreen Mahoney, Mendel Friedman, Mikhail Martchenko Shilman, Kirkwood M. Land

**Affiliations:** 1 Foodborne Toxin Detection and Prevention Research Unit, Western Regional Research Center, Agricultural Research Service, United States Department of Agriculture, Albany, CA 94710, USA; luisa.cheng@usda.gov (L.W.C.); kathy.chan@usda.gov (K.L.C.); christina.tam@usda.gov (C.C.T.); noreen.mahoney@usda.gov (N.M.); 2 Healthy Processed Foods Research Unit, Western Regional Research Center, Agricultural Research Service, United States Department of Agriculture, Albany, CA 94710, USA; mendel.friedman@usda.gov; 3 Henry E. Riggs School of Applied Life Sciences, Keck Graduate Institute, Claremont, CA 91711, USA; mikhail_shilman@kgi.edu; 4 Department of Biological Sciences, University of the Pacific, Stockton, CA 95211, USA; kland@pacific.edu

**Keywords:** antifungal, *Aspergillus*, *Candida*, *Cryptococcus*, drug repurposing, multidrug resistance, pan-azole resistance

## Abstract

Control of fungal pathogens is increasingly problematic due to the limited number of effective drugs available for antifungal therapy. Conventional antifungal drugs could also trigger human cytotoxicity associated with the kidneys and liver, including the generation of reactive oxygen species. Moreover, increased incidences of fungal resistance to the classes of azoles, such as fluconazole, itraconazole, voriconazole, or posaconazole, or echinocandins, including caspofungin, anidulafungin, or micafungin, have been documented. Of note, certain azole fungicides such as propiconazole or tebuconazole that are applied to agricultural fields have the same mechanism of antifungal action as clinical azole drugs. Such long-term application of azole fungicides to crop fields provides environmental selection pressure for the emergence of pan-azole-resistant fungal strains such as *Aspergillus fumigatus* having TR34/L98H mutations, specifically, a 34 bp insertion into the cytochrome P450 51A (*CYP51A*) gene promoter region and a leucine-to-histidine substitution at codon 98 of *CYP51A*. Altogether, the emerging resistance of pathogens to currently available antifungal drugs and insufficiency in the discovery of new therapeutics engender the urgent need for the development of new antifungals and/or alternative therapies for effective control of fungal pathogens. We discuss the current needs for the discovery of new clinical antifungal drugs and the recent drug repurposing endeavors as alternative methods for fungal pathogen control.

## 1. Introduction

Fungal infections, such as human invasive aspergillosis, candidiasis, or cryptococcosis caused by *Aspergillus*, *Candida*, or *Cryptococcus* species, respectively, are persistent problems since effective antifungal drugs are often limited [1]. In addition to 5-flucytosine (5FC), only three classes of antifungal drugs are currently applied in clinical settings, namely, azoles, polyenes, and echinocandins; azoles and polyenes were introduced before 1980, while the echinocandin drug caspofungin (CAS) was approved for use in clinics after the year 2000 [2]. These are the three drug classes used in clinical routines to treat invasive/systemic fungal infections and, therefore, emerging resistance incidences of fungal pathogens to these drugs make fungal diseases a global human health concern [3]. Immunocompromised people are at risk of developing fungal diseases.

For instance, the yeast pathogen *Candida* species are the most common causative agents of bloodstream infections, linked to high morbidity and mortality. While *Candida albicans* is the most prevalent species infecting humans, cases of non-*albicans* infections are also continuously growing. Of note, the non-*albicans *C. auris** is an emerging yeast pathogen showing severe human infections and multidrug resistance, with up to 323 United States (US) clinical cases reported in 2018 [4]. *C. auris* spreads easily between hospitalized patients or nursing home residents, and it has been classified as an “urgent threat” pathogen according to the United States Centers for Disease Control and Prevention’s (CDC’s) 2019 Antibiotic Resistance Threats Report (ARTR) [4]. Numerous other *Candida* species have also been grouped as “serious threat” pathogens because they cause different types of fungal infections including oral and vaginal infections or severe invasive diseases. Many species of this group are resistant to conventional antifungal drugs, with estimated US hospitalization cases in 2017 of 34,800, resulting in 1700 deaths [4].

In addition, azole-resistant strains of other fungal species, including *Aspergillus fumigatus*, also cause life-threatening infections, especially in high-risk, immunocompromised people. Azoles are broadly used for treating human fungal pathogens, where the mechanism of action is to inhibit cellular lanosterol 14 alpha-demethylases involved in fungal ergosterol biosynthesis. Azoles are also increasingly applied in agricultural fields to prevent or treat phytopathogenic fungal diseases in crops. Currently, more than 25% of total fungicide sales are azoles [5]. This provides environmental selection pressure for the emergence of pan-azole-resistant strains such as *A. fumigatus* having the TR34/L98H mutation [5]. The development of azole resistance due to their increased use in human medicine and agriculture is a public health concern, leading to the placement of azole-resistant *A. fumigatus* on the microorganism watchlist, according to CDC’s 2019 ARTR [4].

The spectrum of activity for the current systemic antifungal drugs, including amphotericin B (AMB), 5FC, fluconazole (FLU), itraconazole (ITR), voriconazole (VOR), posaconazole (POS), isavuconazole (ISA), CAS, micafungin (MICA), or anidulafungin (ANI), have been documented [6,7,8]. In brief, the antifungal spectrum has been determined as follows: *C. albicans, Candida glabrata, Candida parapsilosis,* and *Candida tropicalis* (AMB, 5FC, FLU, ITR, VOR, POS, ISA, CAS, MICA, and ANI); *Candida krusei* (AMB, 5FC, ITR, VOR, POS, ISA, CAS, MICA, and ANI); *Candida lusitaniae* (5FC, ITR, VOR, POS, ISA, CAS, MICA, and ANI); *A. fumigatus* (AMB, ITR, VOR, POS, ISA, CAS, MICA, and ANI); *Cryptococcus neoformans* (AMB, 5FC, FLU, ITR, VOR, POS, and ISA); *Fusarium* species (AMB, ITR, VOR, POS, and ISA); *Scedosporium* species (AMB, ITR, VOR, POS, and ISA); *Blastomyces dermatitidis, Coccidioides immitis,* and *Histoplasma capsulatum* (AMB, FLU, ITR, VOR, POS, and ISA); Mucorales (AMB, POS, and ISA). Studies indicated that differential susceptibilities of fungal pathogens to the drugs exist depending on the types of fungi or drugs applied. For example, the yeast pathogens *C. albicans, C. glabrata, C. parapsilosis*, and *C. tropicalis* were susceptible to all antifungal drugs described (AMB, 5FC, FLU, ITR, VOR, POS, ISA, CAS, MICA, and ANI), while the other two *Candida* species (*C. krusei* and *C. lusitaniae*) did not show sensitivity to FLU or AMB, respectively. Of note, except for the *Candida* species and *A. fumigatus*, the other fungi mentioned (namely, *C. neoformans, Fusarium* species, *Scedosporium* species, *Blastomyces dermatitidis, Coccidioides immitis,* and *Histoplasma capsulatum*) did not exhibit susceptibility to the echinocandin drugs (CAS, MICA, and ANI), whereas the azoles POS and ISA were effective against all fungal pathogens described above.

There have also been persistent efforts to improve the efficacy or to reduce the toxicity of conventional antifungal drugs/intervention strategies. For instance, AMB was the first antifungal drug introduced to clinics over five decades ago, for which several types of formulations have been developed with varying toxicity such as infusion-related reactions and nephrotoxicity [9,10]. Three lipid-associated AMB formulations have been developed, which include the AMB lipid complex (AMB-LC), liposomal AMB (L-AMB), and colloidal dispersion of AMB (AMB-CD) with the recommended therapeutic doses of 5, 3–6, and 3–4 mg/kg/day, respectively [9,10].

However, the development of entirely new antifungal drugs is a very expensive and time-consuming process. It is estimated that the overall timelines and costs from new antifungal lead discovery to regulatory approval, especially for those overcoming drug-resistant fungal pathogens, are 10 years and USD >300 million, respectively. In addition, marketing is estimated to cost USD 400 million over the lifespan of a product [11]. Recently, there have been alternative approaches termed antifungal ‘drug repurposing” via which the new utility of various types of marketed, non-antifungal drugs are repositioned as novel antifungal agents. Here, we discuss the current clinical needs for the development of new antifungal therapy, and we comment on the recent antifungal drug repurposing efforts as alternative approaches for the control of fungal pathogens.

## 2. Drug Repurposing Approaches

### 2.1. Repurposing Approaches for the Human Therapeutic Drugs (Non-Antifungals)

Drug repurposing for “medical treatments (other than fungal diseases)” is the repositioning platform of already marketed drugs for treating human diseases to cure new, other types of disorders/health problems such as viral infection, lupus nephritis, and neurodegenerative disease. One of the merits of drug repurposing is that the mechanisms of action, cellular targets, toxicity profile, or safety of the commercial drugs have already been identified, which enables expedited regulatory approval [12,13]. The methodical drug repurposing pipeline largely involves two types of approaches, “experimental testing” approaches such as microtiter plate-based high-throughput screenings and “in silico/computational” approaches that utilize currently available data (omics, drug target, and real-world data, such as the data pertaining to individual’s health status or to the healthcare routinely provided) for the identification of potential new drugs to cure diseases. Systematic drug repurposing needs the accession to and interpretation of molecular, protein, and real-world data, as well as experimental analysis, where data validation in the multicellular or higher organism is the key for industry implementation [12]. In principle, the “antifungal” drug repurposing processes also apply similar approaches for successful drug/compound repositioning (Table S1, Supplementary Materials).

### 2.2. Repurposing Approaches for the New Antifungal Drugs

We performed a PubMed database search in the National Center for Biotechnology Information (NCBI) [14] (https://www.ncbi.nlm.nih.gov/) by applying the keywords “antifungal drug repurposing”, “repurposing [and] in silico [and] fungi (or antifungal, *Candida*, *Cryptococcus*, *Aspergillus*)” plus “antifungal resistance [and] Food and Drug Administration (FDA) (or FDA-approved drug)”, which retrieved a total of 747 articles (accessed on 14 August and 20 October 2020). Each repurposed drug identified was then searched further in PubMed with the search terms “fungi [and] antifungal [and] repurposed drug name (individual)” to provide a comprehensive antifungal spectrum. Articles relevant to the new antifungal drug development are summarized in Table S1 (Supplementary Materials) [15,16,17,18,19,20,21,22,23,24,25,26,27,28,29,30,31,32,33,34,35,36,37,38,39,40,41,42,43,44,45,46,47,48,49,50,51,52,53,54,55,56,57,58,59,60,61,62,63,64,65,66,67,68,69,70,71,72,73,74,75,76,77,78,79,80,81,82,83,84,85,86,87,88,89,90,91,92,93,94,95,96,97,98,99,100,101,102,103,104,105,106,107,108,109,110,111,112,113,114,115,116,117,118,119,120,121,122,123,124,125,126,127,128,129,130,131,132,133,134,135,136,137,138,139,140,141,142,143,144,145,146,147,148,149,150,151,152,153,154,155,156,157,158,159,160,161,162,163,164,165,166,167,168,169,170]. The remaining articles not selected here mainly described (1) antibacterial, antiviral, or antiprotozoal drug development, (2) anticancer drug development, or (3) drug development for other human diseases/conditions including metabolic diseases such as arachidonic acid metabolism, Parkinson’s/neurodegenerative diseases, immune-mediated disease, altered gene expression, and ATP synthase disorder. Six studies adopted “in silico/computational” approaches including experimental validation (Table 1), while the remaining investigations used “experimental testing” approaches, including the utilization of standard antifungal testing protocols such as CLSI M27-A, CLSI M38-A, and EUCAST-AFST E.DEF 7.3 developed by the Clinical and Laboratory Standard Institute (CLSI) [171] or the European Committee on Antimicrobial Susceptibility Testing (EUCAST) [172], respectively, for breakout determination.

## 3. Antifungal Drug Repurposing: Current Measures

### 3.1. In Silico/Computational Repurposing Approaches

In silico/computational repurposing approaches typically use four steps: (1) mining and compilation of pathogen genome data, (2) homology modeling, (3) ligand preparation and molecular docking, and (4) experimental validation in the target pathogens [173]. As described in Table 1, public or nonprofit research sectors such as NCBI, Broad Institute (USA), and the European Molecular Biology Laboratory’s European Bioinformatics Institute (EMBL-EBI) provide updated fungal genome or protein data, which makes the comprehensive mining and compilation of fungal genome/protein data feasible. Protein or chemical databases such as the SWISS-MODEL server, Protein Data Bank (PDB), and PubChem server allow the execution of protein structure homology modeling of drug targets or the computation of 3D structures of candidate compounds. Other online tools, such as the Visual Molecular Dynamics (VMD) program or LigPlot program that automatically plot the protein–ligand interactions, are also currently available (Table 1). The execution of ligand preparation and the molecular docking step rely mainly on the application software, as documented in Table 1.

Noteworthy is the repurposing study performed by de Oliveira et al. [15] targeting the saprobic/dimorphic *Paracoccidioides* species, a causative agent of the systemic mycosis paracoccidioidomycosis, which adopted three additional steps: (a) identification of “orthologs” in different isolates of the target pathogen, (b) identification of “homologs” in the drug–target databases, and (c) selection of essential targets in the model fungus *Saccharomyces cerevisiae* system. The study compiled proteins of three *Paracoccidioides* species (*P. lutzii*, *P. americana*, and *P. brasiliensis*) via the Broad Institute Fungal Genomics Database, followed by the identification of orthologs in *Paracoccidioides* species. The study by de Oliveira et al. resulted in the selection of two anticancer drug candidates as new, repurposed antifungals, where the mode of action was to inhibit the fungal phosphatidylinositol 3-kinase TOR2 (Target Of Rapamycin 2) [15].

The validation of antifungal activity of new, repurposed drugs to achieve more than 99.9% fungal death (breakpoints) requires standard testing methods developed by CLSI [171] or EUCAST [172] (Section 3.2). Of the six in silico/computational studies described in Table 1, two investigations adopted the CLSI protocols while four other studies applied various agar- or liquid-based antifungal assays. It is expected that the number of in silico/computational investigations will increase in the coming years, especially with the increasing numbers of omics, drug target, and chemical structural data being generated. The adoption of standard methods, such as CLSI or EUCAST, is highly desired for the unbiased/repeatable determination of the breakpoints (and, thus, the efficacy) of repurposed antifungal drugs.

### 3.2. Experimental Repurposing Approaches

#### 3.2.1. Standard Dilution Methods: CLSI and EUCAST

The majority of articles (Table S1, Supplementary Materials) performed drug repurposing via experimental testing approaches. These include CLSI, EUCAST, or other microdilution/agar assays such as biofilm bioassay, high-throughput ATP content assay, microdilution, and fluorescent microscopic analysis, metabolism and hyphal inhibitory assays, drug diffusion susceptibility testing, human neutrophils, epithelial cell adhesion and invasion assays, murine model, and macrophage assay, among others.

The standard dilution methods developed by the CLSI and EUCAST quantitatively determine (1) minimum inhibitory concentrations (MICs) and minimum fungicidal concentrations (MFC) of drugs/compounds via the microdilution assay settings, as well as whether the antifungal efficacy of drugs/compounds is fungicidal or fungistatic, where fungicidal indicates a ratio of MFC/MIC ≤4 [211], and (2) the levels and types of drug/compound interactions when two drugs/compounds are co-applied, thus calculating the fractional inhibitory concentration indices (FICI) using MIC values or the fractional fungicidal concentration indices (FFCI) using MFC values; synergism indicates FICI or FFCI values ≤0.5, while indifference indicates FICI or FFCI values >0.5–4 [212].

The other method termed the “disc diffusion” test is a relatively inexpensive assay compared to the standard dilution method, for which a few standard assay protocols have been documented in CLSI. CLSI M44 was validated only for azoles and echinocandins for the isolates of *Candida* species, while CLSI M51-A and supplement M51-S1 qualitatively analyze the efficacy of CAS, triazoles, AMB, etc. [213]. There is a lower agreement between the disc diffusion test results and that of the standard dilution assays, especially in the values from *Aspergillus flavus* (AMB and VOR) and *A. fumigatus* (AMB), suggesting that the standard dilution assay seems useful to determine the interpretative breakpoints for both *Candida* and *Aspergillus* species.

#### 3.2.2. Biofilm Analysis

Studies have shown that many fungi can alternate planktonic (freely floating, homogeneous cells) and sessile (surface-aggregated, heterogeneous cells) growth, which significantly affects fungal pathogenesis and human infection [214,215,216]. The sessile, multicellular communities of fungi, also known as biofilms, are highly structured fungal communities, which are either adherent to biological or physical surfaces, such as oral mucosa, denture acrylic substrates, and catheters, or form aggregates within the protective extracellular matrix (ECM). The majority of clinically important fungi can produce biofilms, which include filamentous fungal pathogens (*Aspergillus*, *Fusarium*, and zygomycetes), yeast pathogens (*Blastoschizomyces, Saccharomyces, Malassezia, Trichosporon, Cryptococcus*, and *Candida* species), and *Pneumocysitis* [214,215,216]. There are also variations in biofilm morphology depending on the types of fungi, namely, (1) *C. albicans* forms complex morphology with blastospores, hyphae, and ECM, (2) *C. neoformans* forms an organized structure having yeast cells with a matrix, (3) *A. fumigatus* forms hyphal cells with ECM, (4) *Trichosporon asahii* forms yeast and hyphal cells with ECM, etc. [216]. Therefore, in addition to the standard in vitro testing for the planktonic cells, such as CLSI or EUCAST assays, a highly reproducible microtiter plate-based colorimetric measurement determining metabolic activities of the pathogen’s biofilm are also used [217].

In general, the development cycle of a fungal biofilm consists of initial adhesion, colonization, proliferation with ECM production, biofilm maturation, and dispersion [214,215,216]. Various environmental factors induce surface attachment and biofilm formation of fungi, including the flow of body fluids (urine, blood, saliva, mucus), pH, temperature, and host immune factors, whereby biofilms protect fungi from the harsh environments including antifungal drugs (e.g., ECM shields fungal cells from drugs and reduces drug penetration), chemical and physical stress, etc., or enable a community-coordinated gene regulation or metabolism [214,215,216].

Biofilm-forming fungal infections are very difficult to treat, which often involve increased drug-resistance phenotypes [218]. In particular, the triazoles and traditional formulations of polyene drugs are considered inactive against fungal biofilms [219]. Differential antifungal activity of drugs has also been documented against biofilms from different fungal pathogens. For instance, echinocandins and AMB lipid formulations exhibited in vitro and in vivo antifungal activities against *C. albicans* biofilms, while other fungal biofilms, such as *A*. *fumigatus* or *C. auris* biofilms, were resistant to echinocandin drugs including CAS [214,219].

#### 3.2.3. Phenotypic Variability of Infecting Fungi: Conidia, Hyphal, Yeast, and Filamentous Growth

In addition to biofilm formation, the phenotypic variability of fungi could also play an important role in clinical outcomes of therapeutic interventions, including the repurposed drugs/compounds (Table 2). In *C. albicans*, the morphological switch from yeast cells to hyphae (filamentous forms) serves as a crucial virulence factor, which promotes infection and invasion in hosts. There was also a positive correlation between the level of azole resistance and the capability to form a hyphal structure; under hypha-inducing conditions, only the *C. albicans* resistant to azoles could form hyphae while the susceptible isolates could not [220]. In the antifungal drug screenings, the manganese nitrosyl [Mn(PaPy_3_)(NO)](ClO_4_) ({Mn-NO}), a biocompatible NO-donating reagent that delivers NO under visible light, has been determined more effective against the hyphal form of *C. albicans*, when compared to the yeast cells [221]. Triclosan has also been used in oral hygiene products with a broad-spectrum antimicrobial activity. Of note, in *C. albicans*, triclosan at subinhibitory concentrations antagonized the antifungal activity of the azole drug FLU, which was specific under hypha-inducing conditions [222]. This antagonism could be due to the membranotropic characteristic of triclosan and also the unique composition of hyphal membranes [222]. Meanwhile, in *A. fumigatus*, the conidia and hyphal forms of fungal fragments were equally susceptible to the AMB and azole drugs, while hyphal clumps were only susceptible to the relatively high concentrations of AMB [223].

#### 3.2.4. Animal Model Systems

Animal model systems (mammalian and nonmammalian models) are important components for antifungal drug development/discovery including the validation of repurposed drugs [224] (Table 3).

Mammalian models are represented by murine, rat, guinea pigs, and rabbits, including both naïve and compromised mice, whereby the antifungal activity of the repurposed drugs can be examined for pharmacokinetics (PK) (e.g., tissue distribution, excretion), pharmacodynamics (PD), immune responses elicited by fungal pathogens, and vaccination attempts [224,225] (see [138] in Table S1, Supplementary Materials). There are various animal models and infection routes, together with various immune suppression regimens. Examples include, but are not limited to, drug screening murine model, murine neutropenic thigh model for determining PK/PD of antifungal drugs, murine model for testing mucocutaneous candidiasis, diabetic murine model of disseminated mucormycosis, murine pulmonary mucormycosis, cyclophosphamide/cortisone immunocompromised murine model of pulmonary mucormycosis, persistently neutropenic rabbit model for investigating acute, invasive pulmonary aspergillosis (IPA), and persistently granulocytopenic rabbit model for characterizing the efficacy of L-AMB against IPA [224,225]. It is important to note that animal testing should be compliant with animal welfare regulation, including Institutional Animal Care and Use Committee (IACUC) review, for all proposed animal experiments [226].

Nonmammalian models currently consist of *Drosophila melanogaster* (fruit fly), *Caenorhabditis elegans* (free-living nematode) and *Galleria mellonella* (wax moth). Examples include biofilm formation in *D. melanogaster*, melanization and toxicity testing in *G. mellonela*, and slow and fast killing testing in *C. elegans*, among others. [227]. When compared to the mammalian models, the nonmammalian models are considered affordable and easy to handle (see [154] in Table S1, Supplementary Materials). While studies have shown that many data from nonmammalian models are in parallel with those obtained from mammalian models, some results did not correlate well. The major drawback of nonmammalian models lies in their unsuitability for microbial vaccination, colonization assessment, challenge research, and immune response. Accordingly, mammalian models are considered to better represent the human condition during the efficacy assessment of the repurposed drugs [224], as also described in Section 4.1.

There have been several types of candidate drugs used for antifungal repurposing (Table S1, Supplementary Materials). These include antipsychiatric, estrogen modulator, antidepressant, antiplatelet aggregation, enzyme (serine palmitoyl-transferase) inhibitor, anticardiovascular, antiarthritis, antistroke, antiatherosclerosis, anticancer, and anthelmintic drugs. However, the use of various drug libraries in the antifungal drug repurposing process is also increasing recently; drug libraries have been prepared/preserved by either public or commercial institutes/vendors, as shown in Table 4.

### 3.3. Synergism between Repurposed Agents and Conventional Antifungals

Combination therapy in controlling fungal pathogens is defined as a co-application of two or more antifungal drugs to treat fungal infections [230]. Combination therapy has been developed on the basis that co-administration of antifungal drugs having different cellular/molecular targets could effectively eliminate fungal pathogens, especially those resistant to conventional drugs. However, the efficacy of combination therapies often varies depending upon the types of drugs co-applied. Although many studies determined better results for fungal pathogen control with drug combinations, other data exhibited no added merit of drug co-treatment over the individual application of each drug alone, which may be associated with drug antagonism [231]. Risk factors also exist during combination therapy, such as multidrug interactions and cytotoxicity.

Studies have shown the synergistic interaction between repurposed agents and conventional antifungal drugs, such as FLU, AMB, or CAS (see Table 5 for a summary). For example, Spitzer et al. determined that the antifungal capacity of chemicals can be systematically enhanced via the combined application of known commercial drugs, such as FLU, with other types of bioactive compounds from drug repurposing [23]. They found that the repurposed compounds did not have to possess potent antifungal activity on their own, but that the compounds potentiated the FLU antifungal activity with considerable species specificity. These synergistic drug combinations were different from the traditional combination therapies mentioned above [23].

Similarly, the anticholesterol drug lovastatin has been repurposed as a synergistic antifungal modulator to the azole drug ITR against the planktonic cells and biofilms of the yeast pathogen *C. albicans*; the lovastatin regulation of the ergosterol biosynthetic pathway has been the proposed mechanism of antifungal action [45]. The antifungal mechanism of lovastatin in *A. fumigatus* also involved ergosterol biosynthesis, which was controlled further by cellular iron homeostasis [65]. In *A. fumigatus*, iron starvation induces the production of the siderophore triacetylfusarinine C (TAFC), for which mevalonate is the key intermediate for the synthesis of both ergosterol and TAFC, the critical virulence factors [65]. Of note, the expression of the enzyme 3-hydroxy-3-methyl-glutaryl (HMG)-CoA reductase (Hmg1), responsible for the production of mevalonate, was increased under iron starvation, while the synthesis of TAFC was reduced following the lovastatin-mediated inhibition of Hmg1 [65].

In our prior study, considerable augmentation of the control of *Cryptococcus* species was achieved by the co-application of repurposed compounds, such as octyl gallate or benzaldehydes, with conventional antifungal agents [232]. The *Cryptococcus* species exhibited higher susceptibility to the inhibition of mitochondrial respiration compared to other yeast pathogens *Candida* species [232]. This “species-specific” enhancement of sensitivity to the co-treatments resulted from the inability of *Cryptococcus* species to produce cellular energy (ATPs) via the fermentation process. Studies by Spitzer et al. mentioned above indicated that the differential susceptibility of pathogens to the newly developed drugs or interventions is triggered by the differences in physiological/genetic settings of test strains, which could result in species-specific antifungal responses.

Related antifungal “chemosensitization” has been developed recently as a new intervention strategy, where co-application of a repurposed compound (chemosensitizer), such as food additives, with conventional drugs enhanced the antifungal efficacy of the co-applied drugs [77,233]. A chemosensitizer causes the target pathogen to be more susceptible to the co-applied conventional drug via the modulation of the pathogen’s defense system, such as the oxidative stress signaling system or cell-wall integrity pathway. Considering that the chemosensitizers could also function as probe-like chemicals by negatively affecting specific cellular targets such as antioxidant systems, types of “drug–compound (repurposed) combinations” enable target-specific control of fungal pathogens, including augmentation of the activity of the echinocandin drug CAS by the cell-wall-targeting octyl gallate [234]. The chemosensitization strategy has been applied further to the development of a high-efficiency drug repurposing protocol that could enhance the sensitivity of target pathogens to the drug candidates, thus reducing time/costs for screening new antifungal drugs, as well as overcoming drug/fungicide resistance of fungal pathogens [104].

## 4. Challenges

### 4.1. Pioglitazone: Needs for In Vivo Drug Validation

Pioglitazone (PIO) has been used as an adjuvant of AMB for the treatment of cryptococcosis. AMB causes excessive generation of reactive oxygen species linked to compromised renal function. PIO is an agonist of peroxisome proliferator-activated receptor γ, which is used to treat type 2 diabetes and is also used as an adjuvant of many drugs triggering side-effects due to its redox-active and anti-inflammatory characteristics [235]. In a murine model, co-application of PIO and AMB exhibited higher efficacy than AMB alone for the inhibition of yeast pathogens, whereby the combination (PIO + AMB) disrupted yeast transmission from the lungs to the brain, which also eliminated yeasts that reached the central nervous system [236]. PIO did not exhibit in vitro antifungal activity, nor did it affect the AMB-mediated fungicidal activity of macrophages; however, PIO as a therapeutic adjuvant counteracted the oxidative bursts after the reduction of the fungal burden, thus relieving the oxidative stress damages to the host (in vivo) and preventing the establishment of meningoencephalitis [236]. The fact that the in vitro antifungal activity of PIO and AMB co-application was determined as “indifferent” while that of in vivo administration increased the survivability of the animals, compared to AMB alone, strongly suggests the importance of the in vivo validation of drug repurposing. The in vitro CLSI or EUCAST testing alone could miss identifying highly effective antifungal adjuvants such as PIO (also observed in other drug developments, personal communication [237], American Chemical Society National Meeting, 2020).

### 4.2. Resistance to Repurposed Drugs/Compounds: Cinnamic and Benzoic Derivatives

The model yeast *Saccharomyces cerevisiae* has been used as a useful screening system for identifying antifungal agents in view that (1) the *S. cerevisiae* genome has been sequenced and well-characterized ([238], accessed 3 September 2020), (2) *S. cerevisiae* gene deletion mutants have been very useful for investigating the mechanisms or target genes of screened leads [239], and (3) many genes in *S. cerevisiae* are homologous to those of fungal pathogens [240]. The antifungal compounds screened via *S. cerevisiae* (wild type or mutants) also exhibited broad-spectrum antifungal activities against pathogenic yeasts (*Candida* and *Cryptococcus* species) and filamentous fungi (*Aspergillus*, *Fusarium*, and *Scedosporium* species) [2].

**Figure 1:**
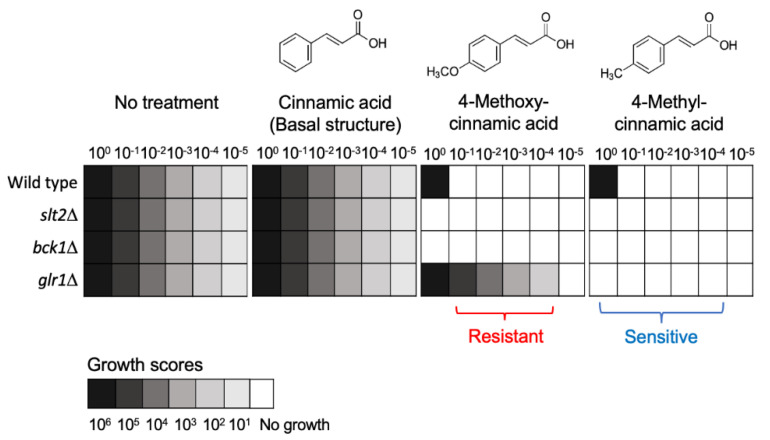
Yeast dilution bioassay showing differential susceptibility of *S. cerevisiae slt2*Δ, *bck1*Δ, and *glr1*Δ mutants to cinnamic acid analogs (0.5 mM) (adapted from [243]). Numbers 10^0^, 10^−1^, 10^−2^, 10^−3^, 10^−4^, and 10^−5^ indicate the cell dilution rate for yeast spotting; growth scores 10^1^, 10^2^, 10^3^, 10^4^, 10^5^, and 10^6^ denote cell numbers which appeared following incubation. *slt2*Δ, mitogen-activated protein kinase (MAPK) mutant; *bck1*Δ, MAPK kinase kinase (MAPKKK) mutant; *glr1*Δ, glutathione reductase mutant.

**Figure 2:**
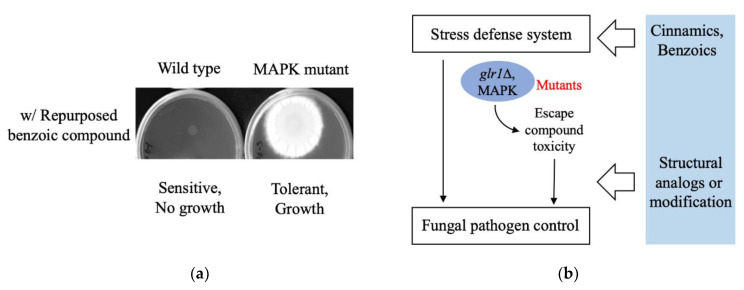
(**a**) *A. fumigatus* MAPK mutant showing tolerance to the repurposed benzoic ingredient (Kim et al., unpublished observation); (**b**) scheme describing structural modifications of cinnamates or benzoates that could overcome the tolerance of *S. cerevisiae glr1*Δ or *A. fumigatus* MAPK mutants, respectively, to the repurposed compounds (see Figure 1 and [243] for cinnamates).

Caution should be exercised during the high-throughput repurposing process so as not to overlook the tolerant response of certain mutants. For instance, cinnamic acids are generally recognized as safe (GRAS) compounds, which have been used as food additives [241]. In recent repurposing studies, cinnamic acid derivatives have been investigated as antifungal alternatives which target fungal cell-wall biosynthesis and integrity [242,243,244]. While the wild type and cell-wall integrity mutants of *S. cerevisiae* showed a sensitive response to the selected cinnamic acids, such as 3- or 4-methoxycinnamic acids, the glutathione reductase mutant (*glr1*Δ) was hyper-tolerant to 4-methoxycinnamic acid when compared to other test strains [243] (Figure 1). This type of hyper-tolerance was eliminated by 4-methylcinnamic acid, which is the structural derivative of 4-methoxycinnamic acid having a deoxygenated *para* methyl moiety (Figure 1). Glutathione reductase is necessary for the reduction of the oxidized glutathione (GSSG) to reduced glutathione (GSH) to maintain cellular redox homeostasis [245]. While the study showed the structure–activity relationship of cinnamic derivatives in targeting fungal cell-wall components where the *para* methyl moiety is critical to overcoming the *glr1*Δ hyper-tolerance to 4-methoxycinnamic acid, the investigation also highlighted the importance of comprehensive determination of the “gene–compound” interaction/response, thus avoiding unfavorable outcomes including fungal tolerance to the repurposed agents (Figure 1; Figure 2b for the scheme) during drug repurposing.

Edible plants including herbs are rich sources of bioactive metabolites that possess various hepatoprotective, antihypertensive, antitumor, or immunomodulatory effects. However, natural ingredients in plant extracts, such as benzoic derivatives, could negatively affect the fungal signaling mutants (for example, *A. fumigatus* antioxidant mitogen-activated protein kinase (MAPK) mutants *sakA*Δ and *mpkC*Δ [246,247]) where fungal MAPK mutants showed tolerance to the benzoic ingredients, while the wild-type strains remain susceptible to the molecules (our unpublished observation; see Figure 2a for fungal bioassay and Figure 2b for the scheme). Collectively, studies proved the significance of “gene–compound” interaction analysis during the preclinical stage of drug repurposing (regardless of the sources of the drugs or compounds whether synthetic, natural, crude extracts, or purified), thus circumventing the unfavorable downside of repurposed drugs.

## 5. Summary

Current antifungal intervention strategies often encounter limited efficiency in controlling fungal pathogens. Infections of the bloodstream or lungs by *Candida* species or the airborne *Cryptococcus*/*Aspergillus* species, respectively, that are resistant to conventional drugs cause serious health issues. Resistance to drugs develops via the repeated usage of antifungal agents over time (acquired resistance), while certain fungal species are intrinsically resistant to the conventional drugs (intrinsic resistance); examples of intrinsic resistance include azole resistance (*C. glabrata*, *C. krusei*, and *C. auris*), echinocandin resistance (*Cryptococcus* and *Fusarium* species), and polyene resistance (*C. auris* and *A. terreus*) [248,249].

Drug repurposing for fungal treatments is an alternative strategy for developing new antifungals. In this paper, two types of drug repurposing approaches were discussed, in silico/computational approaches and experimental approaches. While the majority of drug repurposing studies adopted experimental repurposing platforms, it is expected that the numbers of in silico/computational investigations will be increased in the future considering the increasing numbers of in silico data including omics, drug target, and chemical structural data. The preclinical validation of the efficacy of the repurposed drugs will require the testing of molecules in the model systems with the unbiased determination of breakpoints via the standard microdilution protocols developed by CLSI or EUCAST.

While repurposed drugs could be applied independently as novel antifungal agents for treating fungal pathogens, they can also function as effective synergists/adjuvants in formulations to conventional antifungal drugs such as FLU [23]. In contrast to traditional combination therapy, co-application with a repurposed drug could avoid drug antagonism, multidrug interactions, and cytotoxicity. The One Health approach acknowledges that human, animal, and environmental health is closely linked [250], for which drug repurposing could provide solutions to eliminate resistant fungi such as pan-azole-resistant *Aspergillus* species [251].

In summary, drug repurposing could provide promising alternatives to current antifungal practices. Future inclusion of additional resources, in addition to the one described in this paper, such as the DrugCentral database [252] and Aggregate Analysis of ClinicalTrials.gov (AACT) database [253], would improve the antifungal drug repurposing processes that have the potential to benefit agriculture, food security, and animal and human health.

## Figures and Tables

**Table 1:** Tools and procedures applicable to the in silico/computational antifungal drug repurposing.

Pathogens Steps	*Paracoccidioides* Species	*Candida albicans*	*Candida, Aspergillus*, and *Trychophyton* Species	*Pythium insidiosum*	*Paracoccidioides* Species	*Candida auris*
Mining and compilation of pathogen genome data	Broad Institute Fungal Genomics Database (https://www.broadinstitute.org/fungal-genome-initiative)	The Basic Local Alignment Search Tool (BLASTP), National Center for Biotechnology Information (http://www.ncbi.nlm.nih.gov/BLAST/); CLUSTALW, European Bioinformatics Institute (http://www.ebi.ac.uk/Tools/msa/)	MOE 2014.09 (PDB search module); Clustal Omega tool, UniProt server (http://www.ebi.ac.uk/Tools/msa/clustalo/)	UniProt database (https://www.uniprot.org); Protein Data Bank (https://www.rcsb.org); BLASTP (blast 2.2.28_ program) (https://www.ncbi.nlm.nih.gov)	-	-
Identification of orthologs in pathogen isolates	OrthoVenn server	-	-	-	-	-
Identification of homologs in the drug–target databases	DrugBank; Therapeutic targets database (TTD)	-	-	-	MDL Drug Data Report (MDDR) (BIOVIA and Thomson Reuters); DrugBank (https://www.drugbank.ca/); TargetMol provider (http://targetmol.com/)	-
Selection of essential targets in the *Saccharomyces cerevisiae* system	Database of Essential Genes (DEG)	-	-	-	-	-
Homology modeling	SWISS-MODEL server; Protein Data Bank (PDB); KoBaMIN server; HCC server; MolProbity	SWISS-MODEL server; Ramachandran plot, SAVES (http://services.mbi.ucla.edu/PROCHECK)	AMBER99 (with *S. cerevisiae* chorismate mutase; PDB entry code 4CSM) as a template	PubChem server (http://pubchem.ncbi.nlm.nih.gov/) (3D structure); PreADMET server (https://preadmet.bmdrc.kr/introduction/) (3D structure)	Homology modeling for *Candida albicans* thioredoxin reductase [174]	Cytochrome P450 51 (*CYP51*) modeling [175]
Ligand preparation and Molecular docking	OMEGA v.3.0.0.1 software by OpenEye Scientific [176]; QUACPAC v.1.7.0.2 software by OpenEye Scientific [177]; OEDocking suite v.3.2.0 by OpenEye Scientific [178]; FRED program with the ChemGauss4 score function in the OEDocking suite	Protein Preparation Wizard of Schrodinger’s suite 8.5 (Chimeric 1EA1); Docking between internal ligand (fluconazole) and 1EA1 in Schrodinger’s suite 8.5; GROMACS 5.0 for molecular dynamics simulations with GROMOS9643a1 force field; LIGPREP and MAESTRO (fluvastatin 3D structure); MATLAB version R2015b (plotting and calculations); Dundee Prodrug 2.5; GLIDE 5.0	MOE 2014.09 (Chemical Computing Group Inc., Sherbooke St, Montreal, QC, Canada) software; Leadit 2.1.2 (BioSolveIT GmbH, Germany) software; Gromacs 4.5.5 (Molecular dynamics); PRODRG2 and GROMOS 53A6 force field (Topology); Particle Mesh Ewald (PME) method and linear constraint solver (LINCS); Xmgrace and VMD software	AutoDock Vina program; PyRx suite open-source software version 0.9.7; AutoDock Vina program; Discovery Studio Visualizer version 17.2.0 (Dassault Systemes Biovia Corp.)	Scaffold Hunter program (http://scaffoldhunter.sourceforge.net/) (selection of top ten best compounds from each database); GOLD software (docking simulation against *Candida albicans* thioredoxin reductase); CORINA (three-dimensional models); Visual Molecular Dynamics (VMD) program (http://www.ks.uiuc.edu/Research/vmd/); LigPlot program (https://www.ebi.ac.uk/thornton-srv/software/LIGPLOT/)	Protein preparation wizard (*C. albicans CYP51*); LigPrep module of the Schrodinger suite (sertraline); Schrodinger suite (LLC, New York, NY) (induced fit molecular docking analysis); Glide module (extra precision feature for sertraline)
Experimental validation in the target pathogen	Modified Clinical and Laboratory Standards Institute (CLSI) protocol	Microtiter bioassay	3-(4,5-dimethylthiazol-2-yl)-2,5-diphenyltetrazolium bromide (MTT) microdilution assay	Agar- and broth-based assay	CLSI M27-A3 [171]	Microdilution (killing kinetics)
References	[15,176,177,178,179,180,181,182,183,184,185,186,187,188,189,190,191,192]	[18,193,194,195]	[16,196,197,198,199,200,201,202,203,204]	[17,205,206]	[20,171,174,207,208,209,210]	[21,175]

**Table 2:** Examples of repurposed drugs negatively affecting biofilm formation, hyphal filamentation, or yeast growth in fungi.

Compounds	Fungi	Effects	Ref.
Haloperidol or benzocyclane derivative	*C. albicans,* *C. glabrata,* *C. neoformans*	Inhibition of filamentation, melanin production, and biofilm formation	[78]
Aripiprazole	*C. albicans*	Inhibition of biofilm formation and hyphal filamentation	[152]
Alexidine dihydrochloride	*C. albicans*, *C. auris*, *A. fumigatus*	Antibiofilm activity	[83]
Mefloquine	*C. albicans,* *C. neoformans,*	Inhibition of the expression of virulence traits: filamentation in *C. albicans* and capsule formation/melanization in *C. neoformans*	[155]
Pentamidine, bifonazole, econazole, alexidine, cetylpyridinium chloride, otilonium bromide, benzethonium chloride, niclosamide, disulfiram, temsirolimus	*C. neoformans*	Inhibition of spore germination and yeast growth	[100]
Sulfonamide drugs	*C. albicans*	Inhibition of biofilm	[113]
Miltefosine	*C. albicans*, *C. auris,* *C. dubliniensis*, *C. glabrata,* *C. krusei*, *C. parapsilosis*, *C. tropicalis,* *Sporothrix schenckii*	Inhibition of both planktonic growth and biofilm formation; inhibition of *Coccidioides posadasii* filamentous phase and *Histoplasma capsulatum* filamentous/yeast phases	[116,117,119]
Mebendazole	*C. neoformans*	Antifungal activity against phagocytized *C. neoformans*: affected biofilms and reduced capsular dimensions	[157]
Quinacrine	*C. albicans*	Inhibition of biofilm and inhibition of planktonic growth (alkaline pH) and filamentation	[123]
Auranofin, pyrvinium pamoate, benzbromarone	*C. albicans*	Inhibition of biofilm formation	[124]
Finasteride	*C. albicans*	Inhibition of urinary biofilm formation and filamentation	[164]
Auranofin	*C. albicans*, *Staphylococcus aureus*	Inhibition of *C. albicans* and *S. aureus* (bacterium) mono- and dual biofilm formation	[161]
Panobinostat	*C. albicans*	Inhibition of biofilm, hyphal, and planktonic growth	[143]
Robenidine	*A. fumigatus,* *C. albicans,* *C. neoformans,* *S. cerevisiae*	Inhibition of yeast cell growth, filamentation, and biofilm formation	[167]
*bis*-Biguanide alexidine dihydrochloride	*C. albicans*	Antifungal and antibiofilm activity	[83]
Halogenated salicylanilide, niclosamide	*C. albicans,* *C. auris*	Antifilamentation and antibiofilm activities	[169]
Arachidonic acid	*C. albicans*, *C. parapsilosis,* *C. glabrata,* *C. tropicalis*	Antibiofilm activity	[88]
Aspirin, ibuprofen	*C. albicans,* *Trichosporon asahii*	Antibiofilm and antiplanktonic activity	[60,62]
Nortriptyline	*C. utilis*, *C. krusei*, *C. glabrata*	Antihyphal and antibiofilm activity	[74]
Quinine	*C. albicans*	Antifungal synergy with bicarbonate or hygromycin against biofilm	[125]

**Table 3:** Examples of animal models used in drug repurposing (see also Table S1, Supplementary Materials).

Drug/Compound Repurposed	Animal Model	Fungi	Effect	Reference
Raltegravir	BALB/c mice, male, 6 weeks old	*Paracoccidioides* species	Reduction of the fungal burden, decreased alterations in the lung structure of mice (1 mg/kg of raltegravir)	[20]
Thioridazine	Murine J774 phagocytes	*C. neoformans*	Decreased the intracellular burden of *C. neoformans* (2.7-fold at a concentration 16-fold below the MIC (2 μg/mL))	[75]
Beauvericin	Specific pathogen-free female ICR (Crl: CD-1) mice	*C. albicans* or *C. parapsilosis*	Reduction in tissue damage and inflammatory cell infiltration in kidneys (0.5 mg/kg beauvericin and KET ^1^)	[85]
Beauvericin	BALB/c (inbred) mice, female, 7 weeks old	*C. albicans*	Beauvericin (4 mg/kg) and FLU ^1^ (0.5 mg/kg) combination exhibited a therapeutic benefit	[84]
Pentamidine	C57BL/6J, female, 8 to 10 weeks old	*C. neoformans*	Mice treated prophylactically with pentamidine (for 3 days prior to infection) resulted in a 2-fold-lower fungal burden than the control; minimized lung fungal burden in spore-mediated infections of mice	[100]
Deferasirox	C57BL/6 mice, female, 4 to 6 weeks old; immunosuppression model of murine oropharyngeal candidiasis	*C. albicans*	Preventive deferasirox treatment significantly reduced the fungal burden in tongue tissue	[137]
*N*-Acetylcysteine	C57/BL6 mice, female, 6 to 8 weeks old	*C. gattii*	*N*-Acetylcysteine + AMB ^1^ achieved higher survival than the control and reduced morbidity in murine-induced cryptococcosis; reduced fungal burden in lungs/brain and lower concentrations of proinflammatory cytokines in the lungs	[138]
Cisplatin	BALB/c mice, female, 4 to 6 weeks old	*C. neoformans*	Cisplatin significantly inhibited *C. neoformans* growth in a mouse model	[168]
Panobinostat	*Galleria mellonella* larvae	*C. albicans*	Panobinostat and FLU combination enhanced survival rate of *G. mellonella*	[143]
Pilocarpine hydrochloride	*G. mellonella* larvae	*C. albicans*	Pilocarpine hydrochloride protects *G. mellonella* larvae from *C. albicans-*induced mortality in a dose-dependent manner	[154]
Pitavastatin	*Caenorhabditis elegans* animal model	*C. albicans*	Pitavastatin–FLU combination reduced the biofilm formation of *Candida* species and the fungal burdens in a *C. elegans* infection model	[77]

^1^ Drug abbreviations: amphotericin B (AMB), fluconazole (FLU), and ketoconazole (KET).

**Table 4:** Summary of the drug/compound libraries used in the antifungal drug repurposing (see also Table S1, Supplementary Materials).

Drug, Compound Libraries	Sources	Fungi Tested	References
MDL Drug Data Report (MDDR), DrugBank, TargetMol databases or library (L4200)	BIOVIA and Thomson Reuters https://www.drugbank.ca/, http://targetmol.com/	*C. albicans,* *Paracoccidioides* species	[20,167]
Prestwick Chemical Library	Prestwick Chemical (Illkirch, France) http://www.prestwickchemical.com/	*Candida* species, *Cryptococcus* species, *Saccharomyces cerevisiae,* *Aspergillus fumigatus,* *Fusarium oxysporum,* *Fusarium solani,* *Lichtheimia* species, *Lomentospora prolificans,* *Paecilomyces variotii,* *Rhizopus arrhizus,* *Scedosporium apiospermum*	[23,25,66,75,83,94,96,124,165,228]
Pharmakon1600 drug library	MicroSource Discovery Systems (Gaylordsville, CT, USA) http://www.msdiscovery.com/	*C. albicans,* *C. glabrata* *C. auris*	[68,77,162]
Library of Pharmacologically Active Compounds (LOPAC^1280^)	Sigma-Aldrich (St. Louis, MO, USA) https://www.sigmaaldrich.com/	*Exserohilum rostratum*	[101]
Medicines for Malaria Venture (MMV) Malaria Box	Medicines for Malaria Venture (Geneva, Switzerland) https://www.mmv.org/ [229]	*C. albicans*, *C. gatti,* *C. neoformans*, *L. prolificans,* *Fonsecaea pedrosoi,* *Fonsecaea monophora*, *Fonsecaea nubica*, *Cladophialophora carrionii*, *Phialophora verrucosa*, *Rhinocladiela similis*, *Exophiala jeanselmei* var. *heteromorpha*, *Exophiala dermatitidis*	[91,148]
Screen-Well Enzo library of 640 compounds	Enzo Life Sciences (Farmingdale, NY, USA) https://www.enzolifesciences.com/	*Aspergillus* species, *Candida,* *Cryptococcus deuterogattii,* *Saccharomyces*	[110]
L1300 Selleck library, 1018 United States Food and Drug Administration (FDA)-approved Selleck library	Selleck Chemicals https://www.selleckchem.com/	*A. fumigatus,* *C. gattii,* *C. glabrata,* *C. neoformans,* *Trichophyton rubrum*	[73,100]
Pathogen Box^®^ chemical library	Medicines for Malaria Venture (Geneva, Switzerland) https://www.mmv.org/	*C. auris*	[119]
United States National Institutes of Health/National Cancer Institute (NIH/NCI) compound library	Developmental Therapeutics Program of the NIH/NCI (Rockville, MD, USA) https://dtp.cancer.gov/	*A. fumigatus,* *Candida* species, *C. neoformans*	[127]
1547 or 1581 FDA-approved drug library	Johns Hopkins, USA Johns Hopkins Clinical Compound Library (JHCCL) version 1.0	*C. albicans,* *C. auris,* *C. krusei,* *C. parapsilosis,* *C. tropicalis*	[133,163]
678 Maybridge collection	Thermo Fisher Scientific (Waltham, MA, USA) https://www.thermofisher.com/us/en/home/chemicals/maybridge.html	*C. albicans,* *C. auris*	[169]

**Table 5:** Summary of the drugs and compounds exerting synergism during co-application (see also Table S1, Supplementary Materials).

Repurposed Drugs/Compounds Co-Applied.	Conventional Antifungal Drugs with Synergism
*N*-Acetylcysteine, alexidine dihydrochloride, amiodarone, arachidonic acid, aspirin, beauvericin, bis-biguanide alexidine dihydrochloride, benzocyclane, bromperidol derivative, chenodiol, chlorcyclizine, clomiphene, cloperastine, colistin, l-cycloserine, deferasirox, drospirenon, ebselen, erythromycin, glimepiride, ibuprofen, idoxifene, lovastatin, methylene-idoxifene, miltefosine nisoldipine, nortriptyline, panobinostat, perhexiline, pitavastatin, polymyxin b, promazine, pyrvinium pamoate, quinacrine, quinine, ribavirin, riluzole, sertraline, suloctidil, tamoxifen, thioridazine, thiosemicarbazone, toremifene, trifluoperazine	Azoles (clotrimazole, fluconazole, isavuconazole, itraconazole, ketoconazole, miconazole, posaconazole, voriconazole), echinocandins (caspofungin, anidulafungin, micafungin), polyene (AMB), allylamine (terbinafine)

## Supplementary Materials

The following are available online at https://www.mdpi.com/2079-6382/9/11/812/s1, Table S1: Characteristics of repurposed drugs/compounds for control of fungal pathogens.
