# Supplementary material for: Antifungal Drug Repurposing
Source: Antibiotics (Basel). 2020 Nov 15;9(11):812. doi: 10.3390/antibiotics9110812 (PMC7697925; doi:10.3390/antibiotics9110812)
Supplement: Supplementary file 1 [file antibiotics-09-00812-s001.pdf]

Table S1. Characteristics of repurposed drugs/compounds for control of fungal pathogens. [1]

Compounds	Original functions	Target fungi	Repurposing methods; cellular processes affected	References
A. IN SILICO, COMPUTATIONAL				
Vistusertib, BGT-226	Anti-neoplastic drug candidates	*Paracoccidioides* species	Computational chemogenomics; fungal phosphatidylinositol 3-kinase (TOR2)	[15]
Fluvastatin	Anti-high cholesterol & triglycerides (blood)	*Candida albicans, Candida glabrata, Candida tropicalis, Candida dubliniensis, Candida parapsilosis, Aspergillus fumigatus, Aspergillus flavus, Rhizopus oryzae, Rhizopus microspores, Rhizomucor pusillus, Rhizomucor miehei, Mucor racemosus, Mucor mucedo, Mucor circinelloides, Absidia corymbifera, Absidia glauca, Trichophyton mentagrophytes, Trichophyton rubrum, Microsporum canis, Microsporum gypseum, Paecilomyces variotii, Syncephalastrum racemosum, Pythium insidiosum*	Computational, Docking fluvastatin with cytochrome P450 (CYP51) model; inhibition of growth and biofilm formation	[18,19]
Abscisic acid	Plant chorismate mutase inhibitor	*C. albicans, C. parapsilosis, Aspergillus niger, T. rubrum, Trichophyton mentagrophytes*	Ligand-based virtual screening, homology modelling, molecular docking; chorismate mutase	[16]
Disulfiram	Treatment of alcoholism	*P. insidiosum*, *Candida* species, *Cryptococcus* species, *A. fumigatus, Histoplasma capsulatum*	Homology modeling and molecular docking; putative aldehyde dehydrogenase and urease activities, bind/inactivate multiple proteins of *P. insidiosum*	[17,22]
Raltegravir (MDDR, DrugBank, TargetMol databases)	Antiviral drug	*Paracoccidioides* species	*In silico* ligand-based, molecular docking, murine assay; thioredoxin reductase	[20]

Sertraline	Anti-depression drug	*Candida auris*	Killing kinetics assay, post-antifungal effect; *in silico* docking; inhibit ergosterol biosynthesis	[21]
B. EXPERIMENTAL, DRUG SYNERGISM				
Trifluoperazine, tamoxifen, clomiphene, sertraline, suloctidil, L-cycloserine (Prestwick Chemical Library)	Psychiatric medication, estrogen modulator, antidepressant, anti-platelet aggregation, serine palmitoyl-transferase inhibitor	*Candida* species, *Cryptococcus* species, *Saccharomyces cerevisiae, Lomentospora prolificans, Curvularia lunata, Curvularia geniculata, Curvularia spicifera, Alternaria alternata, Fusarium incarnatum, Fusarium solani, Fusarium verticillioides, Scedosporium boydii, Scedosporium apiospermum, Purpureocillium lilacinum, Paecilomyces variottii, Trichosporon asahii*	[2] CLSI; gene-drug network interactions analysis; species- or genus-specific synergism with FLU, VOR, CAS or AMB; perturbed membrane permeability or inhibited sphingolipid biosynthesis	[23,24,29]
Ebselen (Prestwick Chemical Library)	Anti-cardiovascular disease, arthritis, stroke, atherosclerosis, cancer, etc.	*C. auris*, seven *Candida* species, *P. variotii, Cryptococcus neoformans, Rhizopus arrhizus, A. fumigatus, A. niger, Fusarium oxysporum, F. solani, S. apiospermum, Lomentospora prolificans, T. asahii*	CLSI M27-A3, synergism with FLU (*C. albicans*), VOR (*Fusarium* species) or AMB (*Trichosporon asahii*)	[25-28]
Suloctidil, Pyrvinium pamoate, Ebselen (Prestwick Chemical Library)	Anti-cardiovascular disease, arthritis, stroke, atherosclerosis, cancer, etc., antiplatelet, anthelmintic drugs	*C. albicans, C. auris, Exophiala dermatitidis*	[3] EUCAST, synergism with VOR, ITR, POS, MICO or anidulafungin; interaction with the plasma membrane H+-ATPase, inhibition of membrane trafficking, vacuolar biogenesis, biofilm inhibition & resistance management	[15,30,31,33,34]
Bromperidol derivatives	Antipsychotic drugs	*C. albicans, C. glabrata, Aspergillus terreus*	Checkerboard bioassay, synergism with azoles	[32]
Review: Anti-virulence agents; resistance management, antifungal repurposing	Anti-virulence factors	*C. albicans*	High throughput synergistic screen (HTSS) platform, CLSI M38-A2; targeting virulence factors (e.g., environmental adaptation factors, adhesins, morphogenesis, secreted enzymes, phenotype switching,	[39]

			biofilms), antifungal synergistic drug combination database (ASDCD)	
Polymyxin B, colistin	Antibiotics	Yeasts (*Candida*, *Cryptococcus*, *Rhodotorula*, *Malassezia pachydermatis*, *Exophiala dermatitidis* *S. cerevisiae*), Molds (*Aspergillus*, *Fusarium*, *Scedosporium*, *Lichtheimia*, *Rhizopus*, *Geosmithia argillacea*, *Zygomycetes*), *Batrachochytrium salamandrivorans*	Time-kill study, checkerboard assay; synergism with polyenes & azoles, colistin synergism with CAS, polymyxin B: overcoming multidrug resistance, synergism with Zwiebelane A in fungal vacuole disruption)	[35-38,40-44]
Tamoxifen	Estrogen receptor modulator	*C. neoformans*, *C. albicans*, *Schizosaccharomyces pombe*	CLSI M27-A3; calmodulin inhibition, structural scaffolds (alkylamino group, aliphatic substituent, electronegative substituents), synergism with AMB	[46-48]
Tamoxifen, Toremifene	Estrogen receptor antagonists	*C. neoformans*	CLSI M27-A3, *C. neoformans* gene deletion library assay, macrophage assay, synergism with FLU; prevent calmodulin binding to calcineurin (Cna1), block Cna1 activation	[49]
Lovastatin	Anti-cholesterol drug	*C. albicans*, *C. glabrata*, *Candida tropicalis*, *Candida krusei*, *Candida parapsilosis* Candida utilis, *S. cerevisiae*, *R. oryzae*, *T. mentagrophytes*, *T. rubrum*, *M. canis*, *M. gypseum*, *A. flavus*, *A. fumigatus*, *A. terreus*, *A. niger*, *Paecilomyces variotii*, *Rhizopus stolonifer*, *Rhizopus homothallicus*, *Mucor circinelloides*, *Mucor racemosus*, *Cunninghamella bertholletiae*, *Mortierella wolfii*, *Syncephalastrum racemosu*,	CLSI M-27A, synergism with ITR or AMB; inhibit ergosterol biosynthesis, planktonic cells and biofilms	[45,50-59]

		Mycotypha africana		
Aspirin, Ibuprofen	Anti-inflammatory drug, blood thinners	*C. neoformans, Cryptococcus gattii, C. albicans, C. glabrata, C. krusei C. parapsilosis, C. tropicalis, Candida guilliermondii, T. asahii, Trichophyton mentagrophytes, Epidermophyton floccosum, M. circinelloides*	EUCAST, macrophage test, antibiofilm, synergism of ibuprofen with FLU, ITR, ISA, VOR, POS, CAS or AMB; activation of the high-osmolarity glycerol pathway, reactive oxygen species-mediated membrane damage	[60-64,67,69,70]
Drospirenon, Perhexiline, Toremifene (Pharmakon1600 library)	Anti-anginal, Birth control, Estrogen receptor modulator	*C. albicans, C. glabrata*	Biofilm cell titer blue assay, checkerboard bioassay, synergism with AMB or CAS	[68]
Erythromycin, Riluzole, Nortriptyline, Chenodiol, Nisoldipine, Promazine, Chlorcyclizine, Cloperastine, Glimepiride (Prestwick Chemical Library)	Antibacterial drug, Treatment of high blood pressure, allergy, etc.	*C. neoformans, Candida* species (including *C. utilis, C. krusei, C. glabrata*) *Pythium insidiosum, Mycosphaerella graminicola*	EUCAST-AFST E.DEF 7.3, synergism with AMB; anti-hyphal and biofilm activity	[66,71,72,74]
Eltrombopag (Compound library of 1018 FDA-approved drugs)	Thrombopoietin receptor agonist	*C. neoformans, C. gattii, C. glabrata, T. rubrum*	Susceptibility testing, synergism with the calcineurin inhibitor FK506; calcineurin pathway, lipid biosynthesis, membrane component, transporter genes, capsule & biofilm formation, melanin production, growth ability at 37°C	[73]
Thirty-one drugs/molecules; amiodarone, thioridazine	Anti-psychotic, Anti-depressant, Anti-estrogen, etc.	*C. neoformans, Fusarium culmorum, Fusarium falciforme, Fusarium nelsonii, F. oxysporum, F. solani, F. verticillioides*	CLSI M27-A2, murine phagocyte assay, synergism with FLU, VOR, CAS or AMB; calmodulin inhibition	[75,80]

(Prestwick Chemical Library)				
Amiodarone	FDA approved antiarrhythmic drug	*A. niger*	XTT (2,3-bis(2-methoxy-4-nitro-5-sulfo-phenyl)-2H-tetrazolium-5-carboxanilide) assay, additive to synergistic to AMB, CLO or KET; affects cellular calcium and pH homeostasis	[76]
Pitavastatin (Pharmakon 1600 drug library)	Control of blood cholesterol level	*C. albicans*, *C. glabrata*, *C. auris*	Biofilm inhibition assay, synergism with FLU	[77]
Haloperidol/ Benzocyclane derivative	Efflux pump modulation, antipsychotics	*C. albicans*, *C. glabrata*, *C. neoformans*, *Microsporum canis*, *Malassezia furfur*, *Malassezia pachydermatis*	CLSI M-27A3, synergism with ITR, VOR or FLU; inhibition of filamentation, melanin production and biofilm formation, overexpression of lanosterol 14a-demethylase (CYP51), Efflux pump expression	[78,79,81,82]
bis-Biguanide alexidine dihydrochloride (AXD) (1200 New Prestwick Chemical Library)	Anticancer drug that targets a mitochondrial tyrosine phosphatase	*A. fumigatus*, *C. albicans*, *C. auris*	Microtiter plate assay, synergism with FLU against biofilms; antifungal, antibiofilm activity	[83]
Beauvericin	Antibiotic, insecticidal	*C. albicans*, *C. glabrata*, *C. parapsilosis*, *C. neoformans*, *A. fumigatus*	CLSI M-27A3, synergism with KET, FLU or MICO, cure the murine model of disseminated candidiasis; drug efflux pump modulation, blocking the ATP-binding cassette transporters, elevating intracellular calcium and reactive oxygen species	[84-87,89]
Arachidonic acid	Polyunsaturated fatty acid	*C. albicans* (AMB resistant), *C. dubliniensis* *C. parapsilosis*, *C. glabrata*, *C. tropicalis*	CLSI M27-A, synergism with FLU, TER, CLO or AMB against biofilms;	[88,90]

			increase in production of prostaglandin	
Ribavirin (Prestwick Chemical Library)	A guanosine anti-viral agent against RNA or DNA virus	*Candida* species	CLSI M27-A2, adjunct therapy, synergism with azoles; fungistatic against multidrug-resistant *C. albicans* and fungicidal against *C. parapsilosis*	[94,95]
Auranofin	Anti-rheumatoid, arthritis	*Candida* species, *Cryptococcus* species, *Fonsecaea pedrosoi*, *Fonsecaea monophora*, *Fonsecaea nubica*, *Cladophialophora carrionii*, *Phialophora verrucosa*, *Rhinocladiela similis*, *Exophiala jeanselmei* var. *heteromorpha*, *Exophiala dermatitidis*	CLSI M27-A3, synergism with ITR (*C. carrionii);* Mia40-Erv1 pathway (a disulfide relay system in mitochondria)	[91,92]
Auranofin	Anti-rheumatoid arthritis	*C. albicans*, *C. glabrata*, *C. krusei*, *C. parapsilosis*, *C. neoformans*, *Blastomyces dermatitidis*, *Cladophialophora carrionii*	CLSI M27-A3 (yeast), M38-A2 (filamentous fungi), synergism with ITR	[91,93]
Alexidine dihydrochloride (Prestwick Chemical Library)	Anticancer drug targeting mitochondrial tyrosine phosphatase (mitochondrial apoptosis)	*C. albicans*, *C. auris*, *A. fumigatus*, *A. flavus*, *A. niger*, *Aspergillus calidoustus*, *F. solani*, *F. oxysporum*, *R. oryzae*, *Lomentos poraprolificans*, *Lichtheimia corymbifera*	CLSI M27-A3, 384-well plates high throughput assay, potentiation of FLU and AMB, antibiofilm activity	[83,96]
Pentamidine, Bifonazole, Econazole, Cetylpyridinium chloride, Alexidine, Otilonium bromide, Benzethonium chloride, Niclosamide, Temsirolimus, Disulfiram (L1300 Selleck Library)	Helminth infection treatment, Anti-cancer, Irritable bowel syndrome treatment, etc.	*C. neoformans*, *C. albicans*, *A. fumigatus*, *A. flavus*, *F. chlamydosporum*, *F. oxysporum*, *F. proliferatum*, *F. solani*, *F. verticillioides*, *Pneumocystis carinii/jiroveci*	Microfluidic, luciferase-based, mice germination assay, CLSI M27-A3, EUCAST E.DEF 9.3, synergism with VOR or AMB (*Fusarium* species); inhibition of spore germination	[97-100,105]

Bithionol, Tacrolimus, Floxuridine (LOPAC libraries)	Anti-parasitic, Immuno-suppressive, antimetabolite	*Exserohilum rostratum*, *A. fumigatus*, *A. flavus*, *A. nidulans*, *A. niger*, *A. terreus*, *C. neoformans*, *C. tropicalis*, *S. cerevisiae*, *Malassezia furfur*, *Malassezia globose*, *Rhizopus delemar* *R. arrhizus*, *R. microsporus* *Lichtheimia corymbifera*, *Lichtheimia ramosa* *M. circinelloides*, *R. pusillus*	High throughput ATP content assay, synergism of *Tacrolimus* with ISA, ITR, FLU or KET; affects ATP level	[101-104,106,108,109]
Flubendazol, nifedipine, nisoldipine, felodipine (Screen-Well Enzo library of 640 compounds)	Anthelmintic, Anti-hypertensive, Calcium channel blocker	*C. neoformans*, *Cryptococcus deuterogattii*, *Candida* species (including *C. albicans*, *C. glabrata*), *Saccharomyces*, *Aspergillus* species (including *A. fumigatus*, *A. flavus*, *A. niger*)	CLSI M27-A3, synergism with FLU, ITR or AMB	[66,107,110-112,114,115]
Twenty-one sulfonamide drugs	Antibacterial drugs	*C. albicans*	CLSI M27-A3, antibiofilm, synergism with FLU in *C. elegans model*; reversal of azole resistance,	[113]
Iodoquinol, Miltefosine (Pathogen Box® chemical library)	Drug candidates	*C. auris*, *C. albicans*, *C. neoformans*, *C. gatti*, *Cladophialophora carrionii* *Phialophora verrucose*, *Fonsecaea monophora* *Fonsecaea nubica*, *Rhinocladiela similis* *Exophiala jeanselmei* var. *heteromorpha* *Exophiala dermatitidis*, *Lomentospora prolificans*, *Sporothrix schenckii*, *Coccidioides posadasii*, *Histoplasma capsulatum*, *A. fumigatus*, *Aspergillus ustus*, *A. flavus*, *Aspergillus* section *Nigri*, *S. apiospermum*, *F. solani*	CLSI M27-A3, iodoquinol synergism with ITR or TER, miltefosine microemulsion with AMB or encapsulation in alginate, antibiofilm (*S. schenkii*), inhibit *Coccidioides posadasii* (filamentous phase), *Histoplasma capsulatum* (filamentous and yeast phases); miltefosine inhibits both planktonic growth and biofilm formation	[91,116-120]
Quinine	Anti-parasite	*C. neoformans*, *C. albicans*, *A. fumigatus*, *Rhizoctonia solani*, *Zymoseptoria tritici*, *Botrytis cinerea*	EUCAST, microtiter plate assay, biofilm inhibition assay; synergism with FLU; synergistic mis-translation in quinine plus hygromycin co-application	[121,125]
Quinacrine	Anti-protozoan drug	*C. albicans*, *C. neoformans*	CLSI M27-A3, Antibiofilm assay;	[122,123]

			synergism with CAS & AMB, antibiofilm activity via vacuolar alkalinization, endocytosis inhibition; impaired filamentation	
Pyrvinium pamoate, Benzbromarone, Auranofin (Prestwick Chemical Library)	Antiseptic, anti-inflammatory, anthelmintic, uricosuric drug, etc.	*C. albicans*, *Exophiala dermatitidis*	Antibiofilm assay via XTT assay; affects biofilm formation, pyrvinium pamoate synergism with POS, ITR, VOR (*E. dermatitidis)* & interference of metal homeostasis (*C. albicans*)	[31,33,124]
NSC319726 (Thiosemicarbazone) (NIH/NCI compound library)	Anti-cancer drug	*Candida* species,*A. fumigatus, A. flavus, C. neoformans*, *Paracoccidioides brasiliensis, Fusarium* species	CLSI M-27A, Drop plate assays, E-tests; ergosterol biosynthesis & ribosomal biogenesis inhibition, synergism with azoles and CAS, antiaflatoxigenic	[126-130,134]
Mycophenolic acid, Disulfiram, Fluvastatin, Octodrine, etc. (1581 FDA approved drug Library)	Immune-suppression, deterrent of alcohol consumption, antihyperlipidemic, decongestant, etc.	*C. albicans, C. neoformans, Trichophyton* species, *A. niger, A. flavus, Aspergillus brasiliensis*	E-test, drug diffusion susceptibility testing, checkerboard assay; synergism with AMB inhibits nucleotide biosynthesis	[131-133,135]
Deferasirox	Iron chelator	*C. albicans, Pythium insidiosum, A. fumigatus, R. oryzae*	Human neutrophils, epithelial cell adhesion & invasion assays; greater susceptibility to oxidative stress, synergism with MICA (*P. insidiosum*), deferasirox improved POS activity with pulmonary mucormycosis	[136,137,139]
N-acetylcysteine (NAC)	Anti-asthma drug	*C. neoformans, Scedosporium aurantiacum, Scedosporium boydii, Pseudallescheria angusta, Pseudallescheria ellipsoidea*	CLSI M38-A2, murine model, macrophage assay; NAC synergism with AMB, TER, decreased capsule size, zeta potential, superoxide dismutase activity, lipid peroxidation; reduced fungal burden in lungs & brain and concentrations of pro-inflammatory cytokines in the lungs	[138,140]

Clioquinol, Alexidine dihydrochloride, Hexachloro-phene, Thonzonium bromide (Prestwick Chemical Library)	Anti-protozoal, Anti-bacterial drug, Cationic detergent (zinc chelator)	*Aspergillus* species (including *A. terreus*), *Fusarium* species (including *F. solani*), *Scedosporium/Lomentospora*, *Rhizopus microsporus*, *Lichtheimia* species (Multidrug resistant), *C. albicans*, *C. glabrata*, *C. parapsilosis*, *C. auris*, *C. tropicalis*, *C. guilliermondii*, *T. harzianum*	CLSI M38-A; synergism with POS	[83,96,141,144]
Panobinostat (FDA-approved)	Pan-histone deacetylase inhibitor, anti-tumor agent	*C. albicans*	CLSI M27-A3, biofilm, hyphal and planktonic growth inhibition, *Galleria mellonella* infection model, synergism with FLU; metacaspase activation (apoptosis)	[143]
C. EXPERIMENTAL, DRUG/COMPOUND ALONE				
Ebselen	Anti-cardiovascular disease, arthritis, stroke, atherosclerosis, cancer, etc.	*C. tropicalis*, *C. albicans*, *C. parapsilosis*	CLSI M27-A3, biopolymeric encapsulation	[145]
Ebselen	Anti-cardiovascular disease, arthritis, stroke, atherosclerosis, cancer, etc.	*C. albicans*, *C. glabrata*, *C. tropicalis*, *C. parapsilosis*, *C. neoformans*, *C. gattii*	CLSI M-27A3, *C. elegans* infection assay, *S. cerevisiae* haplo-insufficiency validation; depletes intracellular glutathione levels; reactive oxygen species production	[142]
Review: Adjuvants (Plant extracts, essential oils, peptides, Drospirenon, Perhexiline, Toremifene, etc.)	Menopausal hormone therapy, Prophylactic antianginal agent, Anti-cancer	Yeast (including *C. albicans*, *C. glabrata*, *C. neoformans*) and filamentous fungal pathogens	Antibiofilm tests, CLSI M27-A3; affects iron & calcium homeostasis, calcineurin & calmodulin, serotonin reuptake, anti-inflammation, histone deacetylase, efflux pump, ABC & MFS transporter, biofilm formation, heat shock protein 90 (Hsp90)	[49,68,147]
4-[6-[[2-(4-aminophenyl)-3H-benzimidazol-5-	Anti- *Plasmodium* drug	*C. gatti*, *C. neoformans*, *C. albicans*, *L. prolificans*	Microdilution and fluorescent microscopic analysis; localization to the nuclei,	[148]

yl]methyl]-1H-benzimidazol-2-yl]aniline (Malaria Box)			apoptosis-like cell death	
Phenothiazines (Trifluoperazine)	Antipsychotic drugs	*Cryptococcal* meningitis, *Candida* species (including *C. albicans*, *C. parapsilosis*, *C. tropicalis*), *Pseudallescheria* species, *Scedosporium* species, *R. microspores* var. *rhizopodiformis* *R. oryzae*, *Rhizopus schipperae* *Saksenaea vasiformis*, *R. miehei* *R. pusillus*, *A. corymbifera*, *Torulopsis glabrata*	CLSI M27-A3, checkerboard bioassays; calmodulin antagonism, modulating undesired neurological effects	[57,140,146,149,150]
Aripiprazole	antipsychotic drug	*C. albicans*	Microtiter plate biofilm inhibition assay, metabolism & hyphal inhibitory assays; biofilm and hyphal inhibition	[152]
Mefloquine	Antimalarial drug	*C. albicans*, *C. glabrata*, *C. auris*, *C. neoformans*, *A. fumigatus*, *S. cerevisiae*	CLSI M27-A3, time-kill assay; interfere with mitochondrial, vacuolar function and filamentation (virulence factor)	[155]
Theophylline (THP)	Respiratory drug	*C. albicans* and non-albicans	CLSI M27-A3; membrane damage, inhibit malate synthase & isocitrate lyase (glyoxylate cycle)	[151]
Pilocarpine hydrochloride	Muscarinic receptor agonist	*C. albicans*	Biofilm viability & cell morphology bioassay, *Galleria mellonella* larvae assay; inhibition of *C. albicans* filamentation and regulation of cellular immunity.	[154]
Flubendazole	Treatment of neglected tropical disease; onchocerciasis	*C. neoformans*	CLSI M27-A2, EUCAST EDef 7.2; binding of flubendazole to cryptococcal β-tubulin	[107]
Oxyclozanide	Anthelmintic	*C. albicans*	Liquid bioassay;	[153]

			uncoupling the mitochondrial electron transport, perturbing mitochondrial membrane potential	
Ebsulfur, Ebselen	Antibacterial drugs	*Candida* species (*C. albicans, C. glabrata, C. krusei, C. parapsilosis*), *Aspergillus* species (*A. flavus, A. nidulans, A. terreus*)	Broth dilution assay, time-kill assay; induction of reactive oxygen species	[159]
Pyrazole derivative	p21-activated protein kinase inhibitor	*F. oxysporum,F. graminearum, Phytopthora* species,*Myrothecium roridum, Helminthosporium maydis, C. albicans, C. krusei, C. tropicalis*	*In vitro* agar assay	[156,158]
Mebendazole	Anti-helminthic	*C. neoformans, C. gatti*	Microtiter bioassay; antifungal activity against phagocytized *C. neoformans*, affected biofilms	[157]
Review: 17-AAG, Hsp 90 inhibitors	Anti-cancer drug	*Candida* species, *Aspergillus* species	Biofilm inhibition assay; combination with azole without host toxicity	[160]
Finasteride	5-α-reductase inhibitor; treatment of benign prostatic hyperplasia	*C. albicans*	Urinary biofilm assay using XTT; inhibition of filamentation	[164]
Thirty-two compounds (Prestwick Chemical Library)	Human hormone, etc.	*C. albicans*	High-throughput, multiplexed flow cytometry & dose-response assay; induction of efflux pump Cdr1p	[165]
Auranofin	Anti-rheumatoid arthritis	*C. albicans*, *Staphylococcus aureus* (bacterium)	CLSI M100–S25; anti-biofilm XTT assay; inhibition of *S. aureus* and *C. albicans* mono- and dual biofilm formation	[161]
Twenty compounds (Pharmakon 1600 compound library)	Alcoholism medication, Anti-depressant, Anti-amoeba	*C. albicans*	CLSI M27-A, biofilm inhibition assay, adherence inhibition screen; blocking calcium channels, inhibition of a selective serotonin reuptake & azole-based proton pump inhibitor	[162]
Lopinavir (1547 FDA-approved	HIV protease inhibitor	*C. auris, C. albicans, C. krusei,*	CLSI M27-A3, *C. elegans* infection model;	[163]

drug library)		*C. parapsilosis*, *C. tropicalis*	interfere with the glucose permeation and ATP synthesis	
Pterostilbene, procyanidin, dichlorophen, tea polyphenol (FDA-approved)	Human disease	*C. albicans*	CLSI M27-A3; phosphopantetheinyl transferase Ppt2 inhibition	[166]
Robenidine (1068 FDA-approved drug library)	Anticoccidial agent reating coccidian infections of poultry and rabbits	*A. fumigatus*, *C. albicans*, *C. neoformans*, *S. cerevisiae*	Growth curve, biofilm assay; inhibit yeast cell growth, filamentation, biofilm formation, and cell wall integrity pathway	[167]
Deferasirox	FDA-approved iron chelator treating iron overload	*C. albicans*	Immunosuppression model of murine oropharyngeal candidiasis; reduction in survival in neutrophil phagosomes, greater susceptibility to oxidative stress, reduced adhesion to and invasion of oral epithelial cells	[137]
Cisplatin	FDA-approved anti-cancer drug	*C. gattii*, *C. neoformans*	Murine model of disseminated cryptococcosis; cisplatin inhibited Prp8 intein splicing, significantly inhibits the growth of Prp8 intein-containing *C. neoformans* and *C. gattii*	[168]
Halogenated salicylanilide, Niclosamide (678 Maybridge collection)	FDA-approved anthelmintic in humans	*C. neoformans*, *C. albicans*, *C. auris* (multidrug-resistant)	Microtiter plate assay; antifilamentation, antibiofilm activities	[169,170]

[1] Drug abbreviations: amphotericin B (AMB), 5-flucytosine (5FC), fluconazole (FLU), itraconazole (ITR), voriconazole (VOR), posaconazole (POS), isavuconazole (ISA), ketoconazole (KET), miconazole (MICO), clotrimazole (CLO), caspofungin (CAS), micafungin (MICA), anidulafungin (ANI), terbinafine (TER).

[2] CLSI, Clinical & Laboratory Standards Institute.

[3] EUCAST, European Committee on Antimicrobial Susceptibility Testing.
